# Chemical Profile, Antioxidant, Antimicrobial, and Anticancer Activities of the Water-Ethanol Extract of *Pulicaria undulata* Growing in the Oasis of Central Saudi Arabian Desert

**DOI:** 10.3390/plants10091811

**Published:** 2021-08-31

**Authors:** Hamdoon A. Mohammed, Mohsen S. Al-Omar, Riaz A. Khan, Salman A. A. Mohammed, Kamal A. Qureshi, Manal M. Abbas, Osamah Al Rugaie, Essam Abd-Elmoniem, Adel M. Ahmad, Yasser I. Kandil

**Affiliations:** 1 Department of Medicinal Chemistry and Pharmacognosy, College of Pharmacy, Qassim University, Buraidah 51452, Saudi Arabia; m.omar@qu.edu.sa; 2 Department of Pharmacognosy, Faculty of Pharmacy, Al-Azhar University, Cairo 11371, Egypt; 3 Department of Medicinal Chemistry and Pharmacognosy, Faculty of Pharmacy, JUST, Irbid 22110, Jordan; 4 Department of Pharmacology and Toxicology, College of Pharmacy, Qassim University, Buraidah 51452, Saudi Arabia; m.azmi@qu.edu.sa; 5 Department of Pharmaceutics, Unaizah College of Pharmacy, Qassim University, Unaizah 51911, Saudi Arabia; ka.qurishe@qu.edu.sa; 6 Department of Medical Laboratory Sciences, Faculty of Allied Medical Sciences, Al-Ahliyya Amman University, Amman 19328, Jordan; m.abbas2@ammanu.edu.jo; 7 Department of Basic Medical Sciences, College of Medicine and Medical Sciences, Qassim University, Unaizah 51911, Saudi Arabia; o.alrugaie@qu.edu.sa; 8 Department of Plant Production and Protection, College of Agriculture and Veterinary Medicine, Qassim University, Qassim 51452, Saudi Arabia; ruessam2@yahoo.com; 9 Department of Pharmaceutical Analytical Chemistry, Faculty of Pharmacy, South Valley University, Qena 83523, Egypt; adelpharma2004@svu.edu.eg; 10 Biochemistry and Molecular Biology Department, Faculty of Pharmacy, Al-Azhar University, Nasr City, Cairo 11231, Egypt; kandil.yasser@azhar.edu.eg

**Keywords:** *Pulicaria undulata*, phenolics, flavonoids, anticancer, antioxidant, antimicrobial, Annexin V, MCF-7, K562, PANC-1

## Abstract

*Pulicaria undulata* (L.) C. A. Mey has multiple uses as part of the traditional medicament, and several biological activities of the plant have been corroborated in the scientific literature. The current work evaluates the phytochemical constituents and biological properties of the water-ethanol extract of the *P. undulata* growing in Qassim, the central arid regions of the Kingdom of Saudi Arabia. Qualitative UPLC-ESIQ-TOF analysis identified 27 compounds belonging to the phenolics, flavonoids, triterpenes, coumarins, and of fatty acids chemical classes. The quantitative analysis exhibited 33.3 mg/g GAE (Gallic Acid Equivalents), and 10.8 mg/g QE (Quercetin Equivalents) of the phenolics and flavonoids in the plant’s concentrated (to dryness) water-ethanol extract. The trace elements analysis of the plant’s dry powder established the presence of copper (20.13 µg/kg), and zinc (68.2 µg/kg) in the higher levels of occurrences. In terms of the antioxidant potential of the plant’s extract, the ferric-reducing, and free-radicals scavenging activities were recorded at 47.11 mg/g, and 19.13 mg/g equivalents of the concentrated to dryness water-ethanol extract of the plant. The water-ethanol extract of *P. undulata* also exhibited antimicrobial activity against the tested Gram-positive bacteria, while no activity was observed against the tested Gram-negative bacteria, or the fungi. The MIC (minimum inhibitory concentration) values were in the range of 49 to 1563 µg/mL, whereas the MBC (minimum bactericidal concentration) values ranged from 49 to 3125 µg/mL, against the tested Gram-positive bacteria. The *P. undulata* water-ethanol extract also exhibited potent cytotoxic effects with the IC_50_ value at 519.2 µg/mL against the MCF-7 breast cancer cell-lines, followed by the anticancer activity of erythroleukemic cell-lines, K562 at 1212 µg/mL, and pancreatic cell-lines, PANC-1, at 1535 µg/mL, as compared to the normal fibroblast cells (4048 µg/mL). The Annexin-V assay demonstrated that, as the *P. undulata* extract’s dose increased from IC_50_ to twice of the IC_50_, the percentage of the necrosis was found to be increased in the late apoptosis stage of the cancer cells. These data confirmed the *P. undulata* extract’s ability to inhibit several human cancer cell lines’ growth in comparison to other local halophytes. The antimicrobial activity of the plant was also confirmed.

## 1. Introduction

*Pulicaria*, a plant genus belonging to the family Asteraceae, comprises over 100 plants species of herbaceous nature [1]. The plants of the genus are mainly concentrated in Southern Europe, the Canary Islands, North Africa, the Mediterranean, and parts of the Asian continent. The plant is well distributed in the dry and salty-soil areas of Egyptian Sinai, Yemen, coastal Algeria, hinterlands of Sudan, rocky regions of Iran, and greener and mountainous areas of Pakistan [2,3,4,5,6,7,8,9,10], including in the Indian plains, Deccan plateau, and Himalayan Terai regions [11]. *Pulicaria* species (i.e., *P. crispa*, *P. arabica*, *P. glutinosa*, and *P. undulata*) are also widely dispersed in peninsular Arabia, together with in the dry, and salty-land areas of the Qassim and its adjoining the central dry regions of the Kingdom of Saudi Arabia [12,13]. 

The central region of the Kingdom of Saudi Arabia has specific climatic conditions. It is characterized by a year-long dry and hot climate, with sandstorms, sparse rains, and high-salt, sandy soil [14,15,16]. The local harsh climatic conditions have immensely impacted the area’s scarce flora and fauna. The ecological factors of climate, soil, and the adjoining habitat’s contribution towards determining the generation of plants’ constituents including the biogenetically produced secondary metabolites, plants’ physiological performance, defense mechanisms, and the effects on biological and medicinal properties of the plants, are well known [17,18,19,20]. 

All the plants of the genus *Pulicaria* that are found in the Kingdom of Saudi Arabia are high salt-tolerant plants, termed halophytes. The halophytes are known to grow in the exceptionally salt-rich soil or waters of high-salinity conditions, including marshy and coastal lands. These plants have adapted to survive in the prevailing dry climate and high-salt soil conditions by implementing several habitat-related, morphological, and biochemical mechanisms essential to deal with these exceptional conditions [21,22,23]. The adopted morphological changes and developed physiological characteristics of these plants help them to survive the demanding dry, and extremely harsh climatic conditions in which they are located. The halophytes, which are challenged by high salt-soil and other environmental factors, also, such as low water availability, dryness, humidity, and several external threats including grazing, adopt to needed morphological alterations, and feasible and competent physiological characteristics to respond to these threats. Also, metabolic and biochemical mechanisms alterations, in total or in part, are adopted to counter the habitat’s tribulations [24,25]. Inherently, the halophytes also neutralize the excessive reactive oxygen species (ROS) produced in response to high-salinity conditions and other environmental stress-factors of the surrounding ecosystem [26,27]. Additionally, previous reports have confirmed that the halophytes biosynthesize enzymes, phytoalexins, and secondary metabolites with a higher antioxidant capacity and in comparatively higher concentrations, as compared to the non-halophytic conditions-growing plants. Several secondary metabolites have been isolated and identified from different species of *Pulicaria* plants. For example, the presence of flavonoids and phenolic acids, sterols, and fatty acids are reported from several *Pulicaria* and other halophytic species [5,28,29,30]. Antioxidant and other physiological and hemostasis-related parameters—specifically in the context of soil, trace elements in the soil, generated salinity stress, habitat adaptation, and production and higher accumulation of phytoconstituents—have been found [31,32,33,34,35]. 

Locally, the plants of the genus *Pulicaria* are well-known as medicinal plants. Ara-bian nomads and herbalists have been reported to frequently use several *Pulicaria* spe-cies as remediation for inflammation, diabetes, and gastrointestinal disorders [2,36,37]. The plants from the genus are also used as tonic, food-preservative, in perfumes, and as part of salad in this region of the world. They are also regularly consumed for other culinary purposes. Records of the antiulcer, antimicrobial, antioxidant, and anticancer bioactivities of *Pulicaria* species are also available [38,39]. 

**Figure 1:**
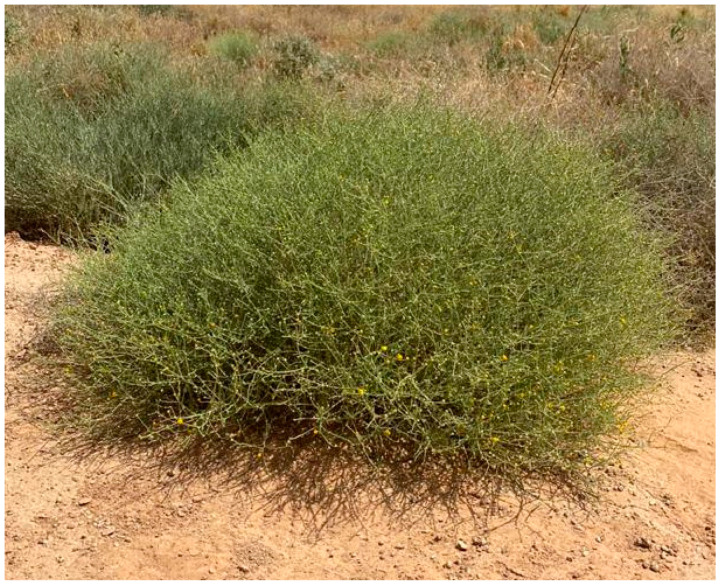
*Pulicaria undulata* (L.) C. A. Mey, size ~2.5 × 5.0 ft. Picture captured by authors at the collection site (latitude 26.348881, longitude 43.766803, and elevation 651 m from the nearest sea level).

*Pulicaria undulata* (L.) C. A. Mey, with the local name “Gethgath” (Figure 1), is a flowering plant, and forms as part of the sunflower plant category, is aromatic in nature. Its volatile constituents are one of the reasons for the plant’s common use in herbal tea, perfumery, and as insect-repellant. The volatile oil compositions of *P. undulata* from the plants growing in different geographic locations [2,3,4,5,6,7] have been investigated in detail. Carvotanacetone, carvacrol, borneol, linalool, and camphor are among the major volatile constituents of the plants’ essential oil. There were noteworthy variations found in the essential oils contents, oils yields, and the oils’ constituents from the plant growing in geographically-different locations for the *P. undulata* species [2,7]. Also, flavonoid class of compounds, mainly of the kaempferol and quercetin glycosidic origins, besides the isoflavone glycosides, have been reported from *P. undulata* [8]. Among other secondary metabolites, the presence of saponins, steroids, triterpenes, coumarins, and tannins have been determined [5]. Traditionally, *P. undulata* is also used as remedy for epilepsy, influenza, the common cold, gastrointestinal disorders, backache, inflamed joints and other body inflammations by the local herbalists and nomads in the northern Sudan [5]. Recently, the cytotoxic effects of the plant extract from *P. undulata*, along with the anticancer activity of its volatile oils, were reported, wherein significant apoptotic effects against HepG2 cell-lines were discovered [2,39]. In addition, the anticancer potentials of the plant’s constituents against several other cancer cell-lines, including multi-drug resistance leukemic cell-lines, were also observed [9,29]. *P. undulata* volatile oils’ and plants’ extracts have also been exhibited to possess significant antioxidant properties, as measured through different assays (i.e., DPPH, ATBS, and FRAP), which supported the traditional herbalists’ claims regarding the plants’ beneficial effects as a nutraceutical, food-additive, tonic, salad, and as traditional medicine for alleviating various symptomatic malfunctioning of the human body [30,40,41]. 

As a halophytic plant, the *P. undulata* is a promising source of essential trace mineral elements, together with some accumulated toxic heavy metals from its elements-rich sandy environment [42,43]. Identification and determination of the abundances of the trace elements concentrations in the plant is also notable from the point of view that the plant is frequently used as a livestock feed. The presence of trace elements is supposed to be complementing to the role of antioxidants in the plant’s defense system against oxidative damage that is brought about by the high salinity of the plant’s environment [44,45]. Accordingly, it was imperative to investigate the elemental compositions of the *P. undulata*. The study was also aimed to investigate the antioxidant potentials, in vitro anticancer, and antimicrobial activities of the *P. undulata* growing in this dry and high-salted central Qassim region of the Kingdom of Saudi Arabia. In addition, qualitative and quantitative analysis were conducted to identify the plant’s phytochemical constituents and measure the phenolics and flavonoids contents in the plant’s extract to further ascertain the quantitative-scale presence of the constituents. 

## 2. Results and Discussion

### 2.1. Identification of Constituents from LC-MS Analysis of the Water-Ethanol Extract

Environmental conditions (i.e., temperature, soil contents, water, and humidity) affect the plants’ growth, its production of secondary metabolic constituents, and its biological activities [46]. The LC-MS profile of the plant *P. undulata*, found in central regions of the Kingdom of Saudi Arabia, was investigated for the first time. From the LC-MS analysis (Table 1) of the water-ethanol extract of the plant, 27 compounds were identified. Some of these identifications were tentative and others were based on comparisons with the available standards. The identified compounds represented different classes of natural products, including fatty and phenolic acids, phenolic glycosides, flavonoids, flavonoid glycosides, and coumarins, as listed (Table 1). 

**Figure 2:**
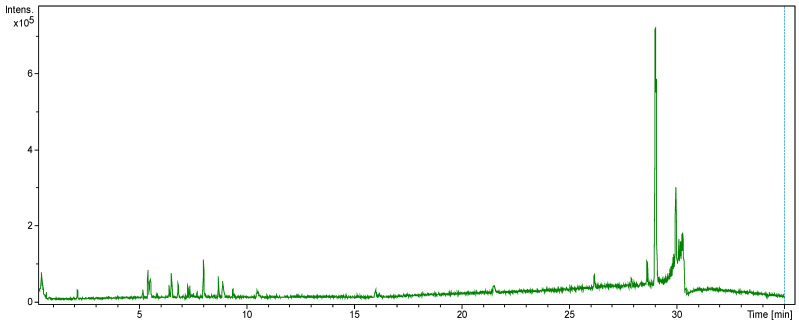
The negative ion mode chromatogram of the water-ethanol extract of *P. undulata*.

**Figure 3:**
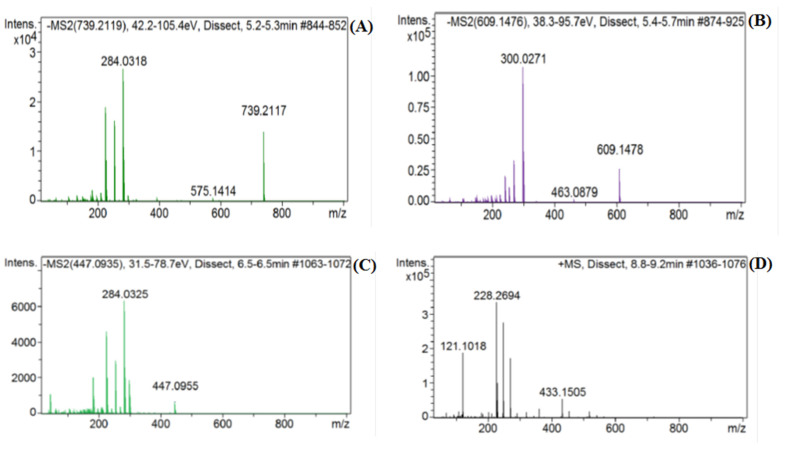
Mass fragment pattern of (**A**) Kaempferol 3-O-rutinoside, (**B**) Rutin, (**C**) Luteolin 7-O-glucoside, and (**D**) Genistin. Mass fragmentation patterns for other compounds in Table 1 are available in the Supplementary File.

The phytoconstituents identities were also confirmed from their mass fragmentation patterns and LC-retention times as compared with the retention times of the utilized standard samples (Supplementary Materials). The chromatogram for the negative ion mode (Figure 2), the positive ion mode (area of interest) chromatograms of the standards, and the mass fragments of the major constituents (Supplementary Materials) were also analyzed, compared, and deliberated upon to confirm the identify the constituents. Among all of the identified constituents, 12 constituents were flavonoids in nature; of which, structurally, seven were flavonols, two flavones, two flavanones, and one of isoflavonoid origins. Some of the identified compounds—quercetin and caffeic acid—were also reported from the *P. undulata* species growing in Egypt [8]. However, all of the other identified constituents reported in the present study have been detected for the first time from this plant. Furthermore, the LC-MS analysis revealed the presence of isoflavonoid glycoside and genistin, together with five other flavonoid aglycones, and six glycosylated flavonoids (Table 1), which amply indicated the plant’s ability to biosynthesize both flavonoids, isoflavonoid aglycones, and glycosides, which was also supported by the earlier report contributed by Hussein et al. [8]. Compared to the total peaks in the negative-ion mode chromatogram, the relative percentages of the identified flavonoids were calculated, and revealed that rutin, hyperoside, luteolin-7-O-glucoside, and quercetin were the major flavonoids at 0.11%, 0.08%, 0.01%, and 0.01%, respectively. The presence of molecular ion peaks, at *m*/*z* 433.1134 [M+H]^+^, 609.1427 [M-H]^−^, 447,0906 [M-H]^−^, and 739.2117 [M-H]^−^ were assigned to the genistin, rutin, luteolin-7-O-glucoside, and kaempferol-3-O-rutinoside, based upon the presence of intense fragment ions peaks of the aglycone fragments at *m*/*z* 271.1314 [M-Glu+H]^+^, 300.0271 [M-rutinoside-2H]^−^, 284.0325 [M-glu-2H]^−^, and 284.0318 [M- neohesperidoside-rhamnoside-2H]^−^, respectively (Figure 3).

Moreover, in addition to these constituents, five phenolics and two coumarins, together with eight fatty acids were also detected from the extract. The presence of phenolic compounds, including flavonoids, seemingly plays a prominent role in the plant’s protection against oxidative stress in its saline habitat and, consequently, a strong antioxidant behavior is observed in the plant’s extract. These compounds are also reported to be beneficial as part of the extract in treating inflammatory disorders [35]. The LC-MS analysis also revealed the similarities in the presence of phytochemical constituents, to a certain extent, between the *P. undulata*, and other *Pulicaria* species (i.e., *P. crispa* and *P. incisa*), which have shown presence of similar phenolics, and flavonoid contents (i.e., quercetin, kaempferol, and caffeic acid) [47]. 

### 2.2. Total Phenolics and Flavonoids Contents

The total phenolics and flavonoids contents in the *P. undulata* were quantitatively determined [2,28]. Phenolics and flavonoids contents were measured as gallic acid, and quercetin equivalents. The presence of 33.31 mg/g of GAE (gallic acid equivalent) and 10.83 mg/g of QE (quercetin equivalents) per gram of the *P. undulata* dry extract were recorded for the total phenolics and flavonoids contents, respectively (Table 2). The results established the presence of higher concentrations of the total phenolics and flavonoids contents of this locally distributed *P. undulata* species than those reported for the other species growing in the Wadi Hagol, Egypt, which showed only 4.13 µg/g, and 1.61 µg/g of the phenolics and flavonoids contents, respectively, per gram of the dry methanolic extract [2,28]. The *Pulicaria undulata* species growing in Lahore, Pakistan, also showed the presence of phenolics and flavonoids contents, together with the presence of alkaloids, saponins, and tannins [30]. 

### 2.3. Trace Elements Analysis

The important roles of trace elements in plants’ and animals’ cellular functions, along with their necessity for human nutrition, are well established [48,49]. In halophytic plants, certain trace elements have been found in high concentrations owing to their mobility from the high salt, and salinity levels of their immediate habitat [50,51,52]. Trace elements (e.g., Fe, Mn, Zn, Cu, and Co) have been analyzed due to their natural abundance in the soils of the Qassim region of Saudi Arabia [53], the habitat this species grows in. Upon analysis, the *P. undulata* collected samples showed the presence of some of these elements (Table 3). 

The trace elements analysis of *P. undulata* confirmed the higher abundance of the elemental iron (Fe = 1008 ± 3.60 µg/kg), as compared to its levels of concentrations in some other halophytes (i.e., *Salsola cyclophylla*, *Salsola imbricata*, *Tamarix aphylla*, *Aeluropus lagopoides*, and *Zygophyllum simplex*) that grow in a similar environment and geographic area [49]. The results also revealed the capability of *P. undulata*, growing in Qassim, Saudi Arabia, to store the copper, and zinc trace elements. The presence of copper and zinc elements in the plant were detected at 20.13 ± 1.69 µg/kg, and 68.2 ± 0.01 µg/kg concentrations, which were nearly two-folds of these elements as reported in the five halophytic plants growing in the same habitat as that of the *P. undulata* [49]. Copper (Cu) as body minerals has significance as its overload disturbs copper homeostasis, and the environmental copper exposure is known to cause oxidative damage, which is also implicated in the abnormal copper metabolism and neurodegenerative alterations due to the excess levels of copper received in the body [54]. The element zinc has an important causative role in the immune system’s performance, and is reported to contribute to supportive antioxidant actions. Zinc is also reported to assist in diabetes management by enhancing insulin secretion and improving the body tissue’s sensitivity toward insulin [55]. The cobalt, manganese, and magnesium elements were also found in the elemental analysis of *Pulicaria undulata*, along with the presence of zinc, iron, and copper (Table 3). The roles of trace elements in human health is well known, and deficiency of cobalt, magnesium, and manganese are leading causes of pernicious anemia, muscle cramps, and impaired skeletal growth, respectively, altering the health situation of humans and animals [56]. 

A comparison of the soil elements from different locations, and climatic zones around the world is presented in Table 4. It distinguishes the comparative values of these elements’ presence in the Qassim soil from other soils and climatic zones. The high elemental composition of the Qassim soil in relation to non-desert and non-halophytic soils from around the world, and the universally acceptable threshold value in relation to ecological and health risks for humans, is presented (Table 4) from the reports [35,57,58,59,60].

### 2.4. Antioxidant Potential of the P. undulata Water-Ethanol Extract

On comparative levels, the antioxidant activity of *P. undulata* volatile oils and extracts have been measured in several preceding works [7,8,30]. The current study demonstrated the antioxidant potential of the water-ethanol extract of the *P. undulata* growing in the central desert lands of Saudi Arabia. The plant’s antioxidant activity tests were performed on a water-ethanol extract using different methods (i.e., DPPH-free radicals scavenging, ferric-ions reducing, and the phosphomolybdate assays) [61]. The plant extract’s ability to reduce the oxidized molybdenum ion was measured as 17.40 mg/g, as the Trolox equivalents per gram of the dried extract of *P. undulata*. The plant also showed potential antioxidant activity as an important scavenging agent by stabilizing the DPPH free radicals at 19.13 mg/g of the Trolox equivalents per gram of the plant dry extract. In addition, the plant extract also demonstrated antioxidant potential through its ability to reduce the ferric ion (FRAP) and chelating of the ferrous ions at 47.11 mg and 8.79 mg of the Trolox and EDTA equivalents, respectively, per gram of the plant’s dry extracts (Table 2). 

The better antioxidant activity was attributed to the phytoconstituents’ antioxidant nature and their higher concentrations; especially in relation to the phenolics, which are abundantly produced owing to harsh variations in the environmental conditions of the plant’s habitat [62,63]. The plant’s LC-MS analysis supported the claimed higher concentrations of the phenolics and flavonoids constituents, especially the phenolics, which is one of the considerable causes of the strong antioxidant power of the local *P. undulata* species. The presence of well-known antioxidant phenolics and flavonoids (e.g., *trans*-ferulic acid, chlorogenic acid, caffeic acid, quercetin, luteolin, rutin, and kaempferol-3-O-rutinoside) [64] were confirmed in the water-ethanol extract of the plant (Table 1). The higher concentrations of these constituents, together with the high levels of the antioxidant activity, and the currently investigated antimicrobial activity of *P. undulata*, together with the presence of several fatty acid constituents (including linoleic acid), has made this Saudi Arabian species, *P. undulata*, a good source of nutrition and a prominent ethnomedicinal herb of the region that is frequently used as part of traditional medicine. A comparative assessment of these characteristics of the locally distributed *P. undulata* in comparison to other locations dispersed species also substantiated the nutritional claims, and food, feed, and ethnomedicinal practices [65,66]. 

### 2.5. Antimicrobial Activity of the P. undulata Water-Ethanol Extract

**Figure 4:**
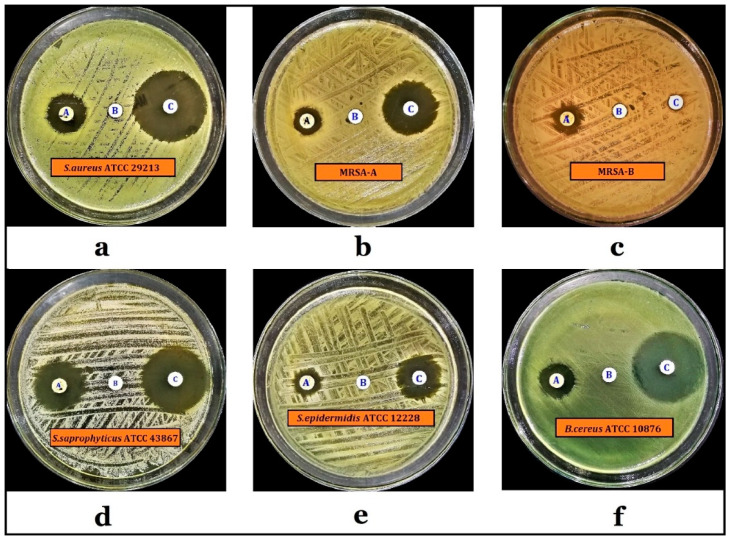
Results of preliminary antimicrobial screenings of *P. undulata* water-ethanol extract against indicator bacteria. A = *P. undulata* water-ethanol extract (2 mg/disc), B = Vehicle solvent (50% *v*/*v* Ethanol), C = Levofloxacin (5 µg/disc). Note: (**a**) *S. aureus* ATCC 29213, (**b**) MRSA-A, (**c**) MRSA-B, (**d**) *S. saprophyticus* ATCC 43867, (**e**) *S. epidermidis* ATCC 12228, (**f**) *B. cereus* ATCC 10876.

The preliminary antimicrobial screening demonstrated that the water-ethanol extract of *P. undulata* possessed substantial antimicrobial activity against the Gram-positive bacteria, including *Staphylococcus aureus* ATCC 29213, *Methicillin-resistant Staphylococcus aureus* (MRSA)-A, *Methicillin-resistant Staphylococcus aureus* (MRSA)-B, *Staphylococcus saprophyticus* ATCC 43867, *Staphylococcus epidermidis* ATCC 12228, and *Bacillus cereus* ATCC 10,876, which were tested during the current study (Figure 4, Table 5). In contrast, no antimicrobial activity was observed against the tested Gram-negative bacteria, and also against any of the tested fungi. However, strong antimicrobial activity against *S. saprophyticus* ATCC 43867 was recorded (i.e., 21.6 ± 0.1 mm), while the weakest antimicrobial activity was recorded against MRSA-A (i.e., 11.5 ± 0.2 mm). The control antibiotic, levofloxacin, inhibited the growth of all the tested Gram-positive bacteria except the MRSA-B, which showed that the tested MRSA-B is resistant to levofloxacin. 

The minimum inhibitory concentration (MIC) and MBC (minimum bactericidal concentration) results demonstrated that the water-ethanol extract of *P. undulata* possessed the MIC values ranging from 49 to 1560 µg/mL, while the MBC values ranged from 49 to 3125 µg/mL against the tested Gram-positive bacteria. The control antibiotic, levofloxacin, inhibited the growth of all the tested Gram-positive bacteria, except MRSA-B at the concentration of 5 µg/mL (Table 6). The presence and alterations in the antimicrobial activity of the plant’s water-ethanol extract is also an indicator of the roles of the constituent phytochemicals produced in consequences of the plant’s response to the habitat, and the environmental conditions [67]. 

### 2.6. Anticancer Activity of the P. undulata Water-Ethanol Extract

**Figure 5:**
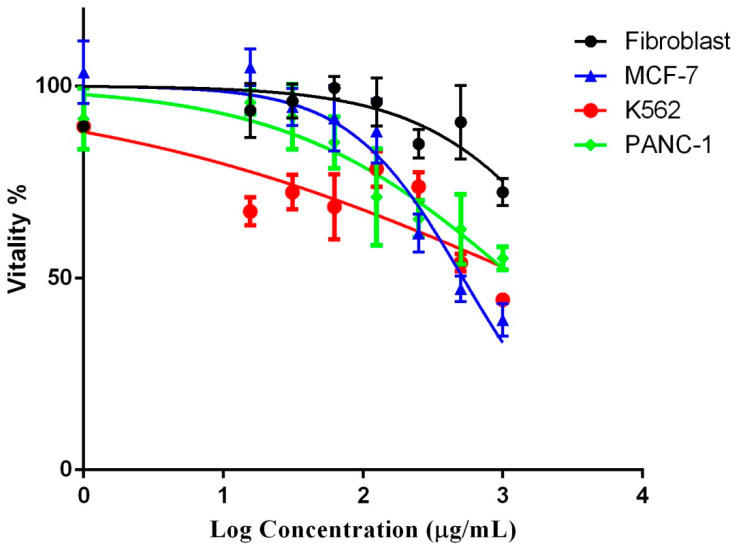
Log dose-response curve of cancer cell-lines compared to the fibroblasts. Values are represented as mean ± SD.

Normal human fibroblasts and three cancer cell-lines, MCF-7, K562, and PANC-1, were cultured, and the antiproliferative potentials of the water-ethanol extract of *P. undulata* were evaluated. The concentrations of 1000 μg/mL were diluted serially down to 6.25 μg/mL and cell vitality, which was found to be dependent on the extract’s dose, was tested (Figure 5). The MCF-7 cell-lines demonstrated the highest percent inhibitions of cancer cells from 39.09 ± 2.44 to 104.87 ± 2.74% with an increasing dose, followed by the proliferation of K562 (44.31 ± 0.65 to 67.35 ± 2.11%), and PANC-1 (55.14 ± 1.75 to 95.75 ± 1.8%) cells. The extract of *P. undulata* demonstrated potent cytotoxic effects, with the lowest IC_50_ value for MCF-7 (IC_50_ 519.2 µg/mL), followed by K562 (IC_50_ 1212 µg/mL), and PANC-1 cells (IC_50_ 1535 µg/mL), in comparison to the normal fibroblast cells (IC_50_ 4048 µg/mL) (Table 7). 

The *P. undulata* water-ethanol extract demonstrated concentration-dependent cytotoxicity for all the cancer cell-lines (Table 8). The K562 cells demonstrated significant cytotoxicity at all the measured concentrations, as compared to the fibroblast. The MCF-7, and PANC-1 cell-lines demonstrated significant cytotoxicity at 250 µg/mL, and 62.5 µg/mL, respectively, compared to the normal fibroblasts. The cell vitality differences remained insignificant when extracts’ concentrations levels were below the 125 µg/mL to 15.625 µg/mL for the MCF-7, and from 31.25 µg/mL to 15.625 µg/mL concentrations for the PANC-1 cell-lines. The MCF-7 cell-lines demonstrated the maximum cytotoxic effects from the lowest to the highest dose levels. Interestingly, in a study by the Jiao group (2017), Cu and Zn presence demonstrated a 20% increase in the cytotoxicity [68]. In the current study, the levels of Cu and Zn in this plant, *P. undulata*, were comparatively high, contrasting with the other halophytes, and the *P. undulata* species inhabiting several different locations. The presence of trace elements may have been supportive in generating significant cytotoxicity. Moreover, some of the identified compounds in *P. undulata* extract (i.e., genistin, 6, 7-dimethoxy coumarin, luteolin, and quercetin) have been previously reported for their in vitro/in vivo anticancer activities [69,70,71,72]. These constituents present in the local *P. undulata* species extract may play a significant role in the antiproliferative effects of the plant’s extract.

**Figure 6:**
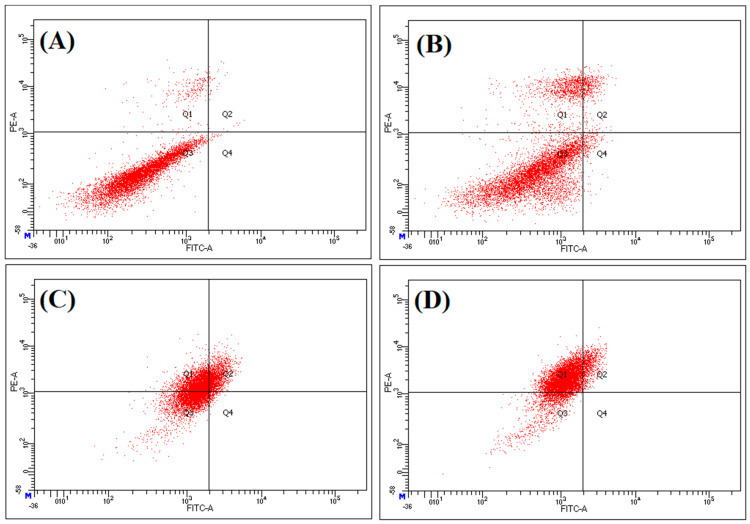
Effects of *P. undulata* water-ethanol extract’s treatment on apoptosis in MCF-7 cell-lines. (**A**) Untreated control, (**B**) 0.5% DMSO, (**C**) IC_50_ of *P. undulata* extract (519.2 μg/mL), (**D**) twice of the IC_50_ of *P. undulata* extract (1038.4 μg/mL).

Moreover, a flow cytometry analysis showed that the *P. undulata* water-ethanol extract induced apoptosis in MCF-7 cell-lines (Figure 6). The cells in the quadrant Q1 and Q2 showed necrosis, as well as late apoptosis, as exhibited by the staining from the Annexin-V-FITC/PI (Fluorescein IsoThioCyanate/Propidium Iodide), and the observance of PI-negative cells, respectively. Annexin-V conjugated to FITC detected the apoptotic cells while PI dyed the membrane’s DNA damaged necrotic, and late apoptotic cells. The Q3 quadrant showed viable cells represented by negative Annexin V-FITC/PI staining, while the Q4 quadrant represented the early apoptotic cells with Annexin V-FITC staining. Both the negative groups’ cells (untreated, and DMSO) were viable (96.1% and 79.6%, respectively), while the viable cells decreased both the IC_50_ and the twice of the IC_50_ (40.3%, and 15.0%) values. The necrotic cells percentage for the water-ethanol extract of *P. undulata* increased from 39.1% to 76.1% for IC_50_ and at twice the IC_50_ value, as compared to the 3.1% and 12.8% for the untreated and DMSO groups (Table 9). Similarly, the early and late apoptotic cells decreased with an increase in the concentrations of the water-ethanol extract of *P. undulata* from IC_50_ to twice the IC_50_ value. These results demonstrated that as the water-ethanol extract of *P. undulata* doses increased from IC_50_ to twice the IC_50_ values, the percent of necrosis increased with a decrease in the viable, early, and late apoptosis of the cells. The early and late apoptosis, along with necrosis, were induced when the cells were treated with dose levels of IC_50_, but early apoptosis was not observed (with reduced late apoptosis). Mainly, the necrosis was observed after the cells were treated with twice of the IC_50_ of the plant’s extract. The DNA damage is not an exclusive feature of apoptosis, and is also observed in necrosis [73,74]. These data confirmed the *P. undulata* extract’s ability to inhibit the growth of the MCF-7 human cancer cell-lines.

## 3. Materials and Methods

### 3.1. Chemicals, Reagents and Materials 

The compounds standards used in the LC-MS analysis were kindly provided by the Center of Naba Hikma Industrial, and Testing Services, Amman, Jordan. DPPH (2,2-diphenyl-1-picrylhydrazyl), ABTS (2,2′-azino-bis(3-ethylbenzothiazoline-6-sulfonic acid), Trolox, quercetin, gallic acid, aluminum chloride, Folin–Ciocalteu reagent, ammonium molybdate, TPTZ (2,4,6-Tris(2-pyridyl)-s-triazine), Ferrozine {3-(2-Pyridyl)-5,6-diphenyl-1,2,4-triazine-p,p′-disulfonic acid monosodium salt hydrate}, and DMSO (dimethyl sulfoxide) were purchased from Sigma Aldrich, MO, USA. MTT (4,5-dimethyl thiazolyl-2,5-diphenyl-tetrazolium bromide) were procured from Promega, Madison, WI, USA, and the TACS^®^ Annexin V–FITC Apoptosis Detection Kit was obtained from R&D Systems, Inc., Minneapolis, MN, USA. Normal human fibroblast, MCF7, PANC-1, and K562 cell-lines were kindly provided by the Al-Ahliyya Amman University, Amman, Jordan. The cell-lines were grown in a humidified 5% CO_2_ atmosphere incubator at 37 °C, and in RPMI 1640 medium with DMEM high glucose (Euroclone, S.p.A) containing 10% FBS (Fetal Bovine Serum), 10 g/L penicillin-streptomycin, and 10 g/L L-glutamine. The microorganisms, *Staphylococcus aureus* (*S. aureus*) ATCC 29213, Methicillin-resistant *Staphylococcus aureus* (MRSA)-A, MRSA-B, *Staphylococcus saprophyticus* (*S. saprophyticus*) ATCC 43867, *Staphylococcus epidermidis* (*S. epidermidis*), *Bacillus cereus* (*B. cereus*) ATCC 10876 ATCC 12228, *Streptococcus pyogenes*-A ATCC 19615, *Streptococcus pneumoniae* ATCC 49619, *Enterococcus faecalis* ATCC 29212, *Bacillus cereus* ATCC 10876, *Escherichia coli* ATCC 25922, *Klebsiella pneumoniae* ATCC 27736, *Pseudomonas aerugenosa* ATCC 9027, *Proteus vulgaris* ATCC 6380, *Proteus mirabilis* ATCC 29906, *Salmonella typhimurium* ATCC 13311, *Shigella flexneri* ATCC 12022, *Candida albicans* ATCC 10231, and *Aspergillus niger* ATCC 6275 were used as test organisms.

### 3.2. Plant Materials and Extraction 

The aerial parts of the plants were collected in March 2019 from the vicinity of the Qassim University campus (collection site: latitude 26.348881, longitude 43.766803, elevation 651 m from the nearest sea level) and identified as *P. undulata* (L.) C. A. Mey by the taxonomist at the College of Agriculture, Qassim University. The collected plant materials (samples were collected from 50 individual plants) were dried in the shade at room temperature (RT) (24 ± 2 °C) for two weeks before grinding. The dried-grinded material (200 g) was macerated with 95% aqueous-ethanol (3 × 1 L) under gentle stirring for 24 h. The combined water-ethanol extract was double filtered, and evaporated to dryness on the Rotavapor < 40 °C to produce a completely dry, thick viscous extract, which was stored at −80 °C and used as such when required. 

### 3.3. Liquid Chromatography-Mass Spectroscopy (LC-MS) Analysis 

A Bruker Daltonics (Bremen, Germany) Impact II ESI-Q-TOF (Electro Spray Ionization-Quadrupole-Time-Of-Flight) System equipped with Bruker Daltonics Elute UPLC system (Bremen, Germany) was used for scanning the extract under 190 and 500 nm range. Specific standards were used to identify the analyte’s retention time in the chromatographic analysis. Accurately, 1 mg of the extract was dissolved in 2.0 mL of DMSO (analytical grade), and the solution was diluted with acetonitrile to 50 mL. The obtained solution was centrifuged at 4000 rpm for 2.0 min, 1.0 mL of the clear extract solution was transferred to the autosampler, and the injection volume was adjusted at 3.0 µL. The instrument was operated using Ion Source Apollo II Ion Funnel Electrospray source. The instrument parameters were adjusted as follows: capillary voltage (2500 V, nebulizer gas (2.0 bar), nitrogen flow (8 L/min), and the dry temperature (200 °C). The mass accuracy was 0.1 Da, the mass resolution was 50,000 FSR (Full Sensitivity Resolution), and the TOF repetition rates were up to 20 kHz. Chromatographic separation was performed on C_18_ RP (Reverse Phase) column (100 × 2.1 mm, 1.8 µm, 120 Å) with Bruker Daltonics (Bremen, Germany) at 30 °C, with the autosampler temperature at 8.0 °C and a total run time of 35 min using the gradient elution. The eluents, A and B, consisted of methanol/5 mM ammonium formate/0.1% formic acid, and water/methanol (90:10)/5 mM ammonium formate/0.1% formic acid, respectively. The software Data Analysis 4.0 (Bruker Daltonics) was used to provide accurate mass data, and molecular formulas of the detected compounds.

### 3.4. Quantitative Measurements of the Total Phenolics and Flavonoids Contents

The phenolics and flavonoids were measured using Folin–Ciocalteu, and aluminum chloride reagents, respectively, as equivalents of the gallic acid and quercetin [40]. For the phenolics quantification, 0.2 mL of the 10% sodium carbonate solution was mixed with 1.6 mL of the extract (0.1 mg/mL in methanol), and 0.2 mL of the diluted Folin–Ciocalteu reagent (1:5, distilled water). The mixture was vigorously mixed, and kept for 30 min at RT before the absorbances were measured at 760 nm. Three independent measurements were recorded, and the total phenolics contents of the *P. undulata* were expressed as gallic acid equivalents (GAE) per gram of the dried extract using the slope equation of the gallic acid calibration curve.

The total flavonoids were measured by mixing 2 mL of the extract (0.1 mg/mL in methanol) and 0.1 mL of the aluminum chloride (10%, aqueous solution) with 0.1 mL of the potassium acetate (0.1 mM) in a test tube. After 30 min of the mixture standing still at RT, the mixture’s absorbances were measured at 415 nm, and the quantified total flavonoids were expressed as quercetin equivalents (QE) per gram of the dried extract from three consecutive measurements. 

### 3.5. Trace Elements Analysis of the P. undulata Water-Ethanol Extract

The dried plants’ (aerial parts) powder of *P. undulata* were used to determine the presence of Fe, Cu, Mn, Co, Mg, and Zn trace elements using ICP-OES (Model iCAP 7400 Duo, serial IC 74DC144208, China) instrument, according to the method described by Johnsson [75]. The plant was dried at 70 °C for two days, grinded and sifted by a stainless-steel mill under 5 mm pore size. As such, the processed plant material (0.5 gm) was digested in a mixture of strong acids, including HNO_3_, HCIO_4_, and H_2_SO_4_ (7:2:1) [49], and the trace element concentrations were measured from the calibration curves prepared for the individual standard elements. The measurements were conducted in triplicate, and were expressed as the mean of the results with their standard deviations.

### 3.6. Antioxidant Activity of the P. undulata Water-Ethanol Extract

#### 3.6.1. Total Antioxidant Capacity 

The plant extract’s antioxidant capacity was measured using the method described by Aroua et al. [76]. The molybdate reagent was prepared by mixing sulfuric acid (0.6 M) and ammonium molybdate (4 mM) in a sodium phosphate buffer (28 mM). Accurately, 3.6 mL of the molybdate reagent was added to 0.4 mL of the extract (containing 200 µg of the extract in methanol), and the mixture was vortexed and kept in a warm water bath for 30 min. The mixture was allowed to cool at RT, and the absorbances were recorded at 695 nm using a spectrophotometer against a blank, which, similarly, was prepared by mixing 0.4 mL of the distilled water with the molybdate reagent. The total antioxidant potential of the extract was calculated as equivalents of the Trolox using the standard calibration curve from the equation (Equation (1)):

y = 0.1954x − 0.1788; R^2^ = 0.9646 (1)

where y is the absorbance of the sample at 695 nm, and x is the concentration of the sample in µg/mL.

#### 3.6.2. DPPH Scavenging Activity 

The ability of the plant extract to scavenge the DPPH-free radicals was determined as Trolox equivalents, according to the method of Shimada et al. [77]. Specifically 1 mL of the extract (containing 200 µg of the extract) in methanol was added to 1 mL of the DPPH (prepared by dissolving 6 mg of the DPPH in 50 mL of methanol). The mixture was vortexed, and kept standing for 30 min in the dark at RT. The absorbances of the mixture were measured at 517 nm by a spectrophotometer against the methanol as a blank. The assay was conducted in triplicate, a standard calibration curve of Trolox against DPPH was generated, and the Trolox equivalents of the extract were calculated from the curve-slope equation. 

#### 3.6.3. Ferric Reducing Antioxidant Power (FRAP) Assay 

The FRAP assay was conducted by following the method of Benzie and Strain [78] with some minor modifications. The working reagent of the FRAP was freshly prepared by mixing one-fold of the TPTZ (2,4,6-Tris(2-pyridyl)-s-triazine, 10 mM prepared in 40 mM HCl, and one-fold of the FeCl_3_ × 6H_2_O (20 mM), with ten-folds of the acetate buffer (300 mM, pH 3.6). Accurately, 2 mL of the FRAP reagent was added to 0.1 mL of the extract (containing 200 µg of the dried extract in methanol), the mixture was incubated for 30 min at RT, and the absorbances were measured at 593 nm. The procedure was conducted in triplicate, and the generated FRAP-Trolox calibration curve was used to calculate the extract activity as mg Trolox equivalents per gram of the used dried extract.

#### 3.6.4. Metal Chelating Activity 

The ability of the plant’s water-ethanol extract to chelate metals, compared to EDTA, was estimated using the method described by Zengin et al. [79]. A mixture of the extract solution (2 mL of methanol containing 200 µg of extract), and Ferrous Chloride (25 µL, 2 mM) was added to 100 µL of Ferrozine to inchoate the color. The mixture’s absorbances were recorded at 562 nm against the blank (2 mL of the extract plus 200 µL of the Ferrous Chloride without Ferrozine). The standard calibration curve of the EDTA was generated and the chelating activity of the extract was calculated in equivalents of the EDTA. 

### 3.7. Antimicrobial Assay of the P. undulata Water-Ethanol Extract

#### 3.7.1. Preliminary Antimicrobial Screenings 

The preliminary antimicrobial screenings of the *P. undulata* plant’s water-ethanol extract were performed by the disc diffusion method [80,81,82]. The plant’s extract was dissolved in 50% *v*/*v* aqueous-ethanol at a concentration of 100 mg/mL. The sterile paper discs (6 mm size) were impregnated with 20 μL of the diluted extract, and each disk had 2 mg of the plant extract under tests on which the antimicrobial activity of the plant extract on different microorganisms were checked. A disk containing 20 μL of 50% *v*/*v* water-ethanol was used as the negative control, while levofloxacin (5 µg/disc) and amphotericin B (100 units/disc) were used as the control antibacterial and antifungal antibiotics, respectively. Each test was performed in triplicate. The inhibitory zones were measured in mm. The results were recorded in mean ± standard deviation (SD). The results obtained from the preliminary antimicrobial activity of *P. undulata* were also analyzed to check whether the tested organisms had statistically different mean values of the antimicrobial activity.

#### 3.7.2. Minimum Inhibitory Concentrations (MIC) and Minimum Bactericidal Concentrations (MBC) 

MIC was conducted following the Resazurin-based micro-broth dilution method while MBC were conducted using the spot inoculation method [83,84,85]. Two-fold serial dilutions of the plant extract with a starting concentration of 100 mg/mL were prepared with 50% water-ethanol and, subsequently, various concentrations of the plant’s water-ethanol extract (0.05 to 25 mg/mL) were prepared in Mueller-Hinton broth (MHB). The prepared concentrations of the plant’s extract were evaluated for their efficacy against selected microorganisms. Levofloxacin was used as a control antibiotic at a concentration of 5 μg/mL.

### 3.8. Cytotoxic Assay of the P. undulata Water-Ethanol Extract

The *P. undulata* extract’s antiproliferative activity was measured against normal human fibroblast. The MCF7, PANC-1, and K562 cell-lines anti-proliferative activities were checked using standard MTT assay on the ability of the mitochondrial dehydrogenase to reduce MTT to a purple formazan product [86]. The cells were suspended at a density of 12–15 × 10^4^ cells/mL in RPMI 1640 media and, exactly 100 µL of each cell type were seeded into each wells of the 96-wells microtiter plate and incubated for 24 h. The *P. undulata* water-ethanol extract was dissolved in 0.5% DMSO and added to the wells in triplicate to a final concentration ranging from 1000 µg/mL to 15.63 µg/mL in a 2-folds serial dilutions (1000, 500, 250, 125, 62.5, 31.25, and 15.63 µg/mL), and incubated at 37 °C, 5% CO_2_ for 24 h. Two controls were used; one contained medium with cells, and the other contained cells plus medium with the vehicle. In addition, the *P. undulata* extract was added without cells to check the effects of the background. Doxorubicin was used as a positive control, and the tests were performed according to the manufacturer’s guidelines. The absorbances were measured at 590 nm using a microplate reader (BioTek, Winooski, VT, USA). 

### 3.9. Annexin V Assay of the P. undulata Water-Ethanol Extract

The MCF7 cells (5 × 10^4^/well) were plated 24 h before the experiment and treated with inhibitory concentrations of 50% of cells (IC_50_) of *P. undulata* for 24 h. Two negative controls—cells without any treatment and cells treated with DMSO—were used. Apoptosis/necrosis were monitored using the TACS^®^ Annexin V–FITC Apoptosis Detection Kit according to the protocol provided by the manufacturer. The percentage of apoptotic/necrotic cells were measured by flow cytometry using a FacsCalibur flow cytometer (BD Biosciences, San Jose, CA, USA).

### 3.10. Statistical Analysis

All descriptive statistics, data analyses, and graphics were performed using GraphPad Prism 8.0.2 (GraphPad Software, San Diego, CA, USA). One-Way ANOVA test combined with Tukey’s analysis method (*post-hoc* analysis) was performed for comparison between means. The significance value was set at *p* < 0.05 [87]. 

## 4. Conclusions

*P. undulata* is among the foremost important medicinal plants that are still in use in traditional systems of medicine in several countries throughout Africa, the Middle East, and Asia. The plant also possesses economic value owing to its uses as a culinary item, green tea, and as part of flavor and perfumery in the regions of its distribution. The current phytochemical and mineral analysis emphasized its nutritional contributions, and pharmacological activities, thereby confirming its folk-lore reputation and traditional uses. The study also provided an insight into the comparative presence and concentrations of phytoconstituents, biological activity, pharmacological efficacy, and antioxidant potentials of the plant growing in this region. The water-ethanol extract of the plant demonstrated cytotoxicity against selected cell-lines, especially MCF7, and exhibited substantial antimicrobials activities. The plant’s water-ethanol extract also scavenged the DPPH free radicals and reduced the oxidized molybdenum and ferric ions that indicated the plant’s strong antioxidant potential. Prospects of potential use of this plant in perfumery, as nutraceuticals in food industries,, and for commercially-viable phytopharmaceuticals preparation for various uses are high. Moreover, further investigations into the bioactivity of the individual constituents, isolated from the plant’s water-ethanol extract and its subsequent fractions, may hold promise for newer molecular leads, and new drug template(s) for further development as part of the drug discovery and development processes.

## Figures and Tables

**Table 1:** Phytoconstituents identified from the *P. undulata* water-ethanol extract by UPLC-ESIQ-TOF technique.

Serial No.	RT (min)	Observed Mass (amu)	Calcd. Mass (amu)	Molecular Formula	Mass Fragments (*m*/*z*)	Compound Class	Compound’s Identity
1.	3.15	179.0725 [M−H]^−^	179.0344	C_9_H_8_O_4_	164, 149, 121, 93	Phenolic acid	Caffeic acid *
2.	3.25	163.03775 [M−H]^−^	163.0395	C_9_H_8_O_3_	120,119, 93,85, 43	Coumarin	Coumaric acid
3.	3.40	471.1875 [M−H]^−^	471.1866	C_22_H_32_O_11_	96, 231, 469	Phenolic glycoside	Eugenol rutinoside
4.	4.50	449.1440 [M+H]^+^	449.1447	C_22_H_24_O_10_	318, 421, 128	Flavanone	Isosakuranin
5.	5.16	193.0551 [M−H]^−^	193.0500	C_10_H_10_O_4_		Phenolic acid	*Trans*-Ferulic acid *
6.	5.30	739.20966 [M−H]^−^	739.2085	C_33_H_40_O_19_	183, 211, 227, 255, 285	Flavonol glycoside	Kaempferol 3-O-rutinoside
7.	5.60	609.14274 [M−H]^−^	609.1455	C_27_H_30_O_16_	255, 271, 301, 302, 463	Flavonol glycoside	Rutin *
8.	5.73	463.0861 [M−H]^−^	463.0876	C_21_H_20_O_12_	201. 215, 227, 255, 271, 300	Flavonol glycoside	Hyperoside
9.	5.95	593.1527 [M−H]^−^	593.1506	C_27_H_30_O_15_	227, 255, 284, 285, 357	Flavonol glycoside	Kaempferol-3-O-rutinoside
10.	6.50	593.14723 [M−H]^−^	593.1506	C_27_H_30_O_15_	301, 286, 271, 255, 227	Flavonol glycoside	3,7-Dirhamnosylquercetin
11.	6.55	447.0906 [M−H]^−^	447.0927	C_21_H_20_O_11_	185, 227, 255, 285	Flavone glycoside	Luteolin 7-O-glucoside
12.	6.82	285.0402 [M−H]^−^	285.0399	C_15_H_10_O_6_	185, 227, 255	Flavone	Luteolin
13.	7.25	301.1050 [M+H]^+^	301.1076	C_17_H_16_O_5_	119, 135, 179	Flavanone	Farrerol
14.	7.91	205.04807 [M−H]^−^	205.0500	C_11_H_10_O_4_	107, 135, 163, 191	Coumarin	Scoparone
15.	8.59	301.0335 [M−H]^−^	301.0339	C_15_H_10_O_7_	271, 255, 243	Flavonol	Quercetin *
16.	8.88	433.1134 [M+H]^+^	433.1134	C_21_H_20_O_10_	271, 250, 231, 221	Isoflavone glucoside	Genistin
17.	10.35	317.0657 [M+H]^+^	317.0661	C_16_H_12_O_7_	137, 228, 274, 301	Flavonol	3-O-Methylquercetin
18.	14.25	285.1098 [M+H]^+^	285.1126	C_17_H_16_O_4_	69, 122, 195	Phenolic acid	Caffeic acid phenethyl ester *
19.	20.50	293.1737 [M−H]^−^	293.1752	C_17_H_26_O_4_	44, 149, 185, 253	Phenolic	6-Gingerol
20.	24.55	227.2008 [M−H]^−^	227.2011	C_14_H_28_O_2_	116, 136	Fatty Acid	Myristic acid
21.	25.71	253.2149 [M−H]^−^	253.2167	C_16_H_30_O_2_	219, 185, 157, 116, 99, 45	Fatty acid	Palmitoleic acid
22.	26.16	279.23045 [M−H]^−^	279.2324	C_18_H_32_O_2_	116, 61, 59, 34	Fatty acid	Linoleic acid
23.	27.91	255.23089 [M−H]^−^	255.2324	C_16_H_32_O_2_	131, 117, 116, 99	Fatty acid	3-Hydroxy dodecanedioic acid
24.	28.23	281.2464 [M−H]^−^	281.2480	C_18_H_34_O_2_	116, 99, 61	Fatty acid	Oleic acid
25.	29.96	283.2620 [M−H]^−^	283.2637	C_18_H_36_O_2_	265, 61, 44	Fatty acid	Stearic acid
26.	30.01	353.34417 [M−H]^−^	353.3419	C_23_H_46_O_2_	255, 116, 89, 74,	Fatty acid	Methyl docosanoate
27.	30.30	423.42272 [M−H]^−^	423.4202	C_28_H_56_O_2_	250, 236, 84, 61	Fatty acid	Octacosanoic acid

RT, Retention Time; Calcd, Calculated; * Compounds identified by comparison with the standards, rest of the identifications are tentative. The mass accuracy was 0.1 Da.

**Table 2:** Total phenolics and flavonoids contents, and antioxidant potential of *P. undulata* water-ethanol extract.

TPC	TFC	TAA	DPPH-SA	FRAP	MCA
33.31 ± 0.46	10.83 ± 0.77	17.40 ± 1.60	19.13 ± 0.01	47.11 ± 4.09	8.79 ± 1.16

All the tests were conducted in triplicate and results were expressed in mean ± standard deviation (SD). TPC, total phenolics contents were calculated in mg/g of gallic acid equivalent; TFC, total flavonoids contents calculated in mg/g of quercetin equivalent; TAA, total antioxidant activity in mg Trolox (6-hydroxy-2,5,7,8-tetramethylchroman-2-carboxylic acid) equivalents per gram of the extract; DPPH-SA, 2,2-diphenyl-1-picrylhydrazyl-scavenging activity in mg Trolox equivalent per gram of the dry extract; FRAP, ferric reducing antioxidant power in mg Trolox equivalent per gram of the dry extract; MCA, metal chelating activity in mg EDTA equivalents per gram of the extract.

**Table 3:** Elemental presence in *P. undulata*.

Trace Elements Concentration in µg/kg * of the Dried Plant Powder					
Iron (Fe)	Copper (Cu)	Magnesium (Mg)	Cobalt (Co)	Manganese (Mn)	Zinc (Zn)
1008 ± 3.60	20.13 ± 1.69	1150.67 ± 7.57	74.00 ± 3.43	99.66 ± 4.40	68.20 ±0.01

* Results were calculated from three measurements and expressed as mean ± SD.

**Table 4:** Elemental composition of different soils.

Trace Elements Concentration in µg/kg of the Soil from Different Locations					
Location	Iron	Copper	Cobalt	Manganese	Zinc
Threshold value *	4600	2–50	1.3	50	200
KSA (Qassim, arid, high salt)	9427	15.9	3.80	195	164
Ghana (tropical green lands)		6.2	1.8		39
Pakistan (Swat area, temperate)		0.44		7.56	0.59
Spain (Alicante region, semi-arid)	15,274	21.6		320	57.8
France (forest sites, temperate)		23.77	1.56		45.01
Egypt (Sinai desert lands)	960	3.03		84	17

* Threshold for ecological and health risks.

**Table 5:** Results of the preliminary antimicrobial screening of *P. undulata* water-ethanol extract.

Microorganisms	Diameters of Zones of Inhibition (mm) *	
	*P. undulata* Plant Extract (2 mg/disc)	Levofloxacin (5 µg/disc)
*S. aureus* ATCC 29213	16.6 ± 0.2	31.7 ± 0.2
MRSA-A **	11.5 ± 0.2	23.4 ± 0.3
MRSA-B **	12.9 ± 0.2	6.2 ± 0.2
*S. saprophyticus* ATCC 43867	21.6 ± 0.1	28.1 ± 0.2
*S. epidermidis* ATCC 12228	11.6 ± 0.2	18.3 ± 0.3
*B. cereus* ATCC 10876	14.9 ± 0.1	28.4 ± 0.2

* All results are in Mean ± SD. Each test was performed in triplicate, ** Clinical isolates.

**Table 6:** Results of MIC, and MBC of *P. undulata* water-ethanol extract.

Microorganisms	*P. undulata* Plant Extract		Levofloxacin (5 µg/mL)
	MIC (µg/mL)	MBC (µg/mL)	
*S. aureus ATCC 29213*	1563	3125	Inhibition
*MRSA-A*	1563	3125	Inhibition
*MRSA-B*	98	195	No inhibition
*S. saptophyticus ATCC 43867*	49	49	Inhibition
*S. epidermidis ATCC 12228*	196	195	Inhibition
*B. cereus ATCC 10876*	49	49	Inhibition

**Table 7:** Antiproliferative IC_50_ values for the *P. undulata* water-ethanol extract.

		IC_50_ (µg/mL)		
	Fibroblast	MCF-7	K562	PANC-1
*P. undulata* water-ethanol Extract	4048	519.2	1212	1535
Doxorubicin	2.975	0.194	0.395	0.158

**Table 8:** Comparative vitality percentage of *P. undulata* water-ethanol extract on the normal fibroblast cells, and MCF-7, PANC-1, and K562 cancer cell-lines.

Concentration (µg/mL)	Fibroblast	MCF-7	PANC-1	K562
1000	72.36 ± 2.05	39.09 ± 2.44 ****	55.14 ± 1.75 **	44.31 ± 0.65 ****
500	90.6 ± 5.53	47.19 ± 1.93 ****	62.69 ± 5.28 ****	53.98 ± 1.31 ****
250	84.91 ± 2.14	61.66 ± 2.85 ****	65.36 ± 2.78 **	73.8 ± 2.11 *
125	95.88 ± 3.61	88.27 ± 4.79 ^ns^	71.12 ± 7.27 ****	78.4 ± 2.69 **
62.5	99.52 ± 1.69	91.63 ± 4.93 ^ns^	85.32 ± 3.87 *	68.59 ± 4.89 ****
31.25	96.09 ± 2.58	94.58 ± 2.78 ^ns^	91.98 ± 4.88 ^ns^	72.36 ± 2.62 ****
15.625	93.62 ± 4.03	104.87 ± 2.74 ^ns^	95.75 ± 1.8 ^ns^	67.35 ± 2.11 ****

Values are represented as mean ± SEM; One-way ANOVA followed by post-hoc test using Tukey’s multi-group comparison was performed: * *p* < 0.05, ** *p* < 0.01, **** *p* < 0.0001 and ns: not significant compared to fibroblast for the measured concentration.

**Table 9:** Annexin V-staining FACS analysis of *P. undulata* water-ethanol extract.

	(A) Untreated	(B) DMSO	(C) IC_50_	(D) Twice of the IC50
Viable	96.1%	79.6%	40.3%	15.0%
Early apoptosis	0.30%	2.60%	2.00%	0.00%
Late apoptosis	0.60%	5.00%	18.6%	8.90%
Necrosis	3.10%	12.8%	39.1%	76.1%

## Data Availability

All data are provided in the manuscript and Supplementary File.

## Supplementary Materials

The following are available online at https://www.mdpi.com/article/10.3390/plants10091811/s1.
